# Two Distinct Determinants of Ligand Specificity in T1R1/T1R3 (the Umami Taste Receptor)*

**DOI:** 10.1074/jbc.M113.494443

**Published:** 2013-11-08

**Authors:** Yasuka Toda, Tomoya Nakagita, Takashi Hayakawa, Shinji Okada, Masataka Narukawa, Hiroo Imai, Yoshiro Ishimaru, Takumi Misaka

**Affiliations:** From the ‡ Department of Applied Biological Chemistry, Graduate School of Agricultural and Life Sciences, The University of Tokyo, Tokyo 113-8657, Japan,; § Primate Research Institute, Kyoto University, Inuyama, Aichi 484-8506, Japan, and; ¶ Japan Society for the Promotion of Science, Tokyo 102-0083, Japan

**Keywords:** Amino Acid, G Protein-coupled Receptors (GPCR), Glutamate Receptors Metabotropic, Mutagenesis in Vitro, Receptor Structure-Function, Taste Receptor, Umami

## Abstract

Umami taste perception in mammals is mediated by a heteromeric complex of two G-protein-coupled receptors, T1R1 and T1R3. T1R1/T1R3 exhibits species-dependent differences in ligand specificity; human T1R1/T1R3 specifically responds to l-Glu, whereas mouse T1R1/T1R3 responds more strongly to other l-amino acids than to l-Glu. The mechanism underlying this species difference remains unknown. In this study we analyzed chimeric human-mouse receptors and point mutants of T1R1/T1R3 and identified 12 key residues that modulate amino acid recognition in the human- and mouse-type responses in the extracellular Venus flytrap domain of T1R1. Molecular modeling revealed that the residues critical for human-type acidic amino acid recognition were located at the orthosteric ligand binding site. In contrast, all of the key residues for the mouse-type broad response were located at regions outside of both the orthosteric ligand binding site and the allosteric binding site for inosine-5′-monophosphate (IMP), a known natural umami taste enhancer. Site-directed mutagenesis demonstrated that the newly identified key residues for the mouse-type responses modulated receptor activity in a manner distinct from that of the allosteric modulation via IMP. Analyses of multiple point mutants suggested that the combination of two distinct determinants, amino acid selectivity at the orthosteric site and receptor activity modulation at the non-orthosteric sites, may mediate the ligand specificity of T1R1/T1R3. This hypothesis was supported by the results of studies using nonhuman primate T1R1 receptors. A complex molecular mechanism involving changes in the properties of both the orthosteric and non-orthosteric sites of T1R1 underlies the determination of ligand specificity in mammalian T1R1/T1R3.

## Teaser

**Results:** The ligand specificity is dependent on a combination of amino acid selectivity at the orthosteric site and receptor activity modulation at the non-orthosteric site.

**Conclusion:** The molecular mechanism underlying the amino acid recognition of T1R1/T1R3 has been elucidated.

**Significance:** This study provides new insights into the molecular mechanisms of the l-Glu-specific response in human T1R1/T1R3.

## Introduction

Umami (amino acid taste), which has been identified as the savory sensation produced by l-glutamate (1), has recently been accepted as one of the five basic tastes. The unique sensory characteristic of umami derives from the synergistic enhancement between l-amino acids and 5′ ribonucleotides, such as IMP and GMP (2). Over the past decade, several candidate umami taste receptors have been proposed, including the heteromeric receptors T1R1/T1R3 (3), mGluR1 (4), mGluR4 (5), taste-mGluR1 (6), and taste-mGluR4 (7, 8). Moreover, several studies have subsequently revealed the crucial roles of T1R1/T1R3 in the perception of l-amino acids and the synergistic effect of IMP using T1R1- and T1R3-KO mice (9–11).

T1R1 and T1R3 are class C G protein-coupled receptors, as are metabotropic glutamate receptors (mGluRs),^2^ the calcium-sensing receptor, the sweet taste receptor component T1R2 (T1R2/T1R3), and others (12). Each of these receptors possesses a large extracellular Venus flytrap domain (VFTD) that is linked to a small extracellular cysteine-rich domain (CRD) and a seven-transmembrane domain (TMD). The structures of the extracellular domains of mGluRs have been determined using x-ray crystallography and indicate that the VFTD consists of two lobes and that the ligand binding site is located in a hinge region between the two lobes (13, 14). Molecular modeling based on the structures of mGluRs and site-directed mutagenesis analysis have also shown that the l-Glu binding site of T1R1/T1R3 lies in the hinge region of the VFTD of T1R1 (15, 16) and that the IMP-binding site lies near the opening of the VFTD of T1R1 (16).

Heterologous expression studies have revealed that mouse T1R1/T1R3 (mT1R1/mT1R3) is broadly activated by most l-amino acids, whereas human T1R1/T1R3 (hT1R1/hT1R3) specifically responds to l-Glu (3, 17). Additionally, the responses of mT1R1/mT1R3 to acidic amino acids are much weaker than those to other amino acids (3). Differences in T1R ligand specificity between species have also been reported for the sweet taste receptor T1R2/T1R3, and multiple ligand binding sites for several sweeteners have been characterized using molecular modeling and site-directed mutagenesis studies of human-rodent (18–21) or human-squirrel monkey (22) chimeric receptors. In contrast, the binding sites for l-amino acids (except for l-Glu) have not been well defined. Additionally, although five residues at the hinge region of hT1R1 have been identified as crucial for l-Glu binding (16), all five residues are conserved between human and mouse T1R1, indicating that the additional residues that are critical for acidic amino acid recognition remain to be identified. Using human-mouse chimeric receptors and point mutants of T1R1/T1R3, we have elucidated the mechanism underlying this difference between species in amino acid recognition.

## EXPERIMENTAL PROCEDURES

### 

#### 

##### Materials

Amino acids were obtained from commercial sources as follows. l-Aspartic acid sodium salt, l-glutamic acid monosodium salt, l-serine, l-lysine monohydrochloride, l-histidine monohydrochloride monohydrate, l-proline, and l-phenylalanine were purchased from Nacalai Tesque (Kyoto, Japan); l-glutamine, l-threonine, glycine, l-alanine, l-valine, l-isoleucine, l-leucine, l-arginine, and l-asparagine monohydrate were obtained from Kanto Chemical (Tokyo, Japan); l-methionine was obtained from Tokyo Chemical Industry (Tokyo, Japan); coelenterazine was purchased from Promega (Madison, WI). The buffer for the luminescence assay comprised 10 mm HEPES, 130 mm NaCl, 10 mm glucose, 5 mm KCl, 2 mm CaCl_2_, and 1.2 mm MgCl_2_ and was supplemented with 0.1% bovine serum albumin (Sigma); the pH was adjusted to 7.4 using NaOH. The ligands were diluted to the desired concentrations in the assay buffer.

##### Constructs for Human, Mouse, and Human-Mouse Chimeric Taste Receptors and Their Point Mutants

hT1R1 (NCBI RefSeq number NM_138697.3), hT1R3 (NM_152228.1), mT1R1 (NM_031867.2), mT1R3 (NM_031872.2), human-mouse T1R1 chimeras, and point mutants of hT1R1 and mT1R1 were constructed by polymerase chain reaction (PCR) using overlapping primers and were subcloned into the pEAK10 expression vector (Edge Biosystems, Gaithersburg, MD) at the AscI-NotI site. The Kozak consensus sequence was introduced upstream of the start codon for efficient translation. To identify the residues that are critical for ligand specificity, we constructed the following chimeric receptors and single-point mutants: hVFTD-mCRD-mTMD T1R1, mVFTD-hCRD-hTMD T1R1, hT1R1 (m.1–143), (m.144–178), (m.179–370), (m.371–380), (m.381–497), (m.179–279), (m.280–329), (m.280–305), (m.306–314), (m.315–329), (m.330–370), (m.373–375), (m.376–380), (m.381–440), (m.452–472), and (m.473–497); hT1R1-S148N, -R151H, -A170E, -E174V, -R281G, -E285R, -T290A, -V298I, -A302D, -L305I, -R307T, -H308Y, -G311N, -R317G, -M320T, -K328Q, -M371T, -A372T, -K377E, -K379G, -S385A, -D435K, -T464E, and -K460E; mT1R1 (h.1–142), (h.143–177), (h.178–369), (h.370–379), (h.380–496), (h.144–151), (h.152–169), (h.170–177), (h.178–218), (h.219–278), (h.380–391), (h.392–422), (h.423–439), and (h.460–471); mT1R1-N149S, -D151N, -H152R, -E171A, -V175E, -I176T, -N277S, -H279Q, -D303A, -T308R, -T321M, -Q329K, -E378K, -G380K, -A386S, -V390A, -E392R, -Q424E, -Y427H, -N430H, -K436D, and -E461K. Moreover, the multiple point mutants hT1R1-A170E/A302D/M320T/K379G and mT1R1-N149S/H152R/E171A/V175E/D303A/T308R/T321M/Q329K/E378K/G380K/K436D/E461K were also constructed.

##### Nonhuman Primate T1R1 Constructs

This study was performed in strict accordance with the recommendations in the Guide for the Care and Use of Nonhuman Primates of the Primate Research Institute, Kyoto University (Version 3, issued in 2010). Genomic DNA was isolated from tongue (macaque) or liver (baboon and squirrel monkey) tissues by digestion with proteinase K. All deduced exons of *Tas1r1* were amplified and sequenced. The PCR primers were designed based on the genome assemblies of the macaque (NC_007858.1), baboon (NW_003871134.1), and squirrel monkey (NW_003943720.1), whose T1R1 loci were annotated using a BLASTN search (23). The amino acid positions Pro-278 and Met-320 in the macaque and Pro-459 in the baboon in this study are distinct from those of the reference sequences because of single nucleotide variations; the Pro-595 codon in the baboon reference sequence also contains a frameshift mutation. The PCR products of each exon were assembled into one full-length sequence using overlapping PCR and were subcloned into the pEAK10 expression vector, as described above for hT1R1 and mT1R1.

##### Luminescence-based Assay for T1R1/T1R3

HEK293T cells were transfected with expression vectors for T1R1, T1R3, rG15i2, and mt-apoclytin-II (24) and maintained at 37 °C under 5% CO_2_ in DMEM supplemented with GlutaMAX (Invitrogen) and 10% dialyzed FBS (Invitrogen) to minimize glutamate-induced desensitization. The transfected cells were seeded on 96-well black-walled CellBIND surface plates (Corning); after 48 h of transfection, a luminescence assay was performed as previously described (25). The response from each well was calculated based on the area under the curve (AUC) and expressed as RLU (relative light units). To examine the EC_50_ values, plots of the amplitudes *versus* concentrations were fitted to the Hill equation. l-Trp and l-Tyr were not assayed because of their insolubility, and l-Cys was not evaluated because of instability in the assay buffer at pH 7.4. The osmotic pressures of the l-Arg and l-His solutions were higher than those of the other amino acid solutions because large amounts of HCl or NaOH were required for the pH adjustment. Statistical analysis was performed using Student's *t* test and one-way ANOVA followed by Dunnett's test using the software Ky Plot version 3.0.

##### Homology Modeling

A homology model of hT1R1 was constructed using MOE (Chemical Computing Group Inc.). In this study we selected the open form of mGluR1 (PDB ID 1EWT) (13) as a template. The model was rendered using Discovery Studio Visualizer (Accelrys).

## RESULTS

### 

#### 

##### Key Domains for Amino Acid Recognition

**FIGURE 1.**
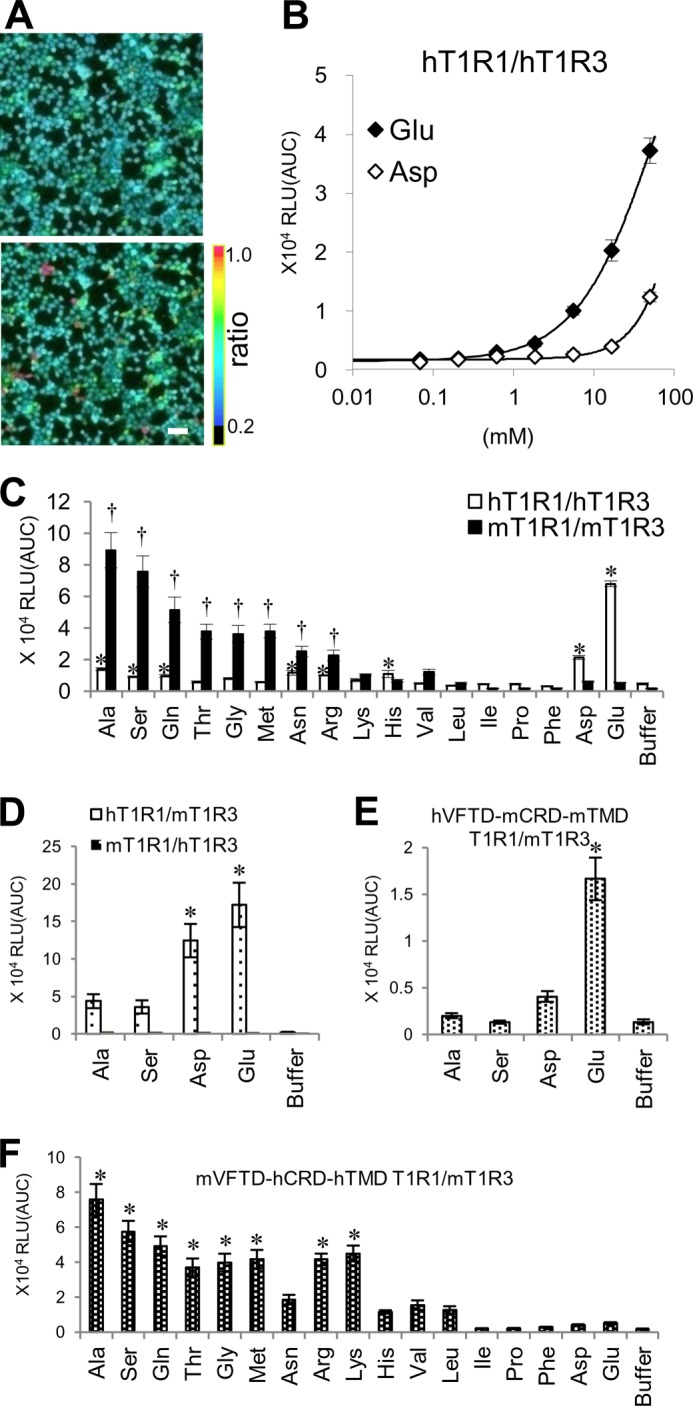
**The domains of T1R1/T1R3 that are critical for ligand specificity.** *A*, the responses of hT1R1/hT1R3 to l-Glu were detected using a Ca^2+^-sensitive fluorescent dye (fura-2). HEK293T cells coexpressing hT1R1/hT1R3 together with rG15i2 were stimulated with 100 mm l-Glu. The figures show representative cell images obtained before (*upper*) and after (*lower*) l-Glu application. The color scale indicates the *F*_340_/*F*_380_ ratio. *Scale bar*, 50 μm. *B*, the dose-response curves of hT1R1/hT1R3 to l-Glu and l-Asp. The changes in intracellular Ca^2+^ mobilization were measured based on the luminescence of mt-clytin-II. The values represent the mean ± S.E. of the RLU (AUC) of four recorded wells. *C*, the response patterns of hT1R1/hT1R3 and mT1R1/mT1R3 to 16 l-amino acids and glycine. HEK293T cells coexpressing hT1R1/hT1R3 or mT1R1/mT1R3 together with rG15i2 were separately stimulated with 50 mm concentrations of each amino acid. *D*, the mixed pairs of human and mouse T1Rs were transiently transfected into HEK293T cells with rG15i2, and the cells were stimulated with 50 mm l-Ala, l-Ser, l-Asp, and l-Glu. *E* and *F*, the cells expressing hVFTD-mCRD-mTMD T1R1 (*E*) or mVFTD-hCRD-hTMD T1R1 (*F*) together with both mT1R3 and rG15i2 were stimulated with 50 mm concentrations of each amino acid. VFTD: residues 1–496 in hT1R1 and residues 1–497 in mT1R1; CRD-TMD: residues 497–841 in hT1R1 and residues 498–842 in mT1R1. The values represent the mean ± S.E. of the RLU (AUC) of 5–6 recorded wells. Significant differences from the response to buffer were analyzed using one-way ANOVA followed by Dunnett's test (*, *p* < 0.05 for hT1R1/hT1R3 (*C*), hT1R1/mT1R3 (*D*), hVFTD-mCRD-mTMD T1R1/mT1R3 (*E*), and mVFTD-hCRD-hTMD T1R1/mT1R3 (*F*); †, *p* < 0.05 for mT1R1/mT1R3 (*B*)).

We previously established a novel high-throughput screening system for the human sweet taste receptor hT1R2/hT1R3 using a luminescence-based assay (25). The assay showed higher sensitivities to intracellular Ca^2+^ changes than the standard fluorescence-based assay when HEK293T cells were transiently transfected with the sweet taste receptor. Therefore, we applied this luminescence-based assay to the umami taste receptor. Consistent with the results of previous studies using fluorescence-based assays (3, 16, 17) (Fig. 1*A*), the responses to l-Glu were detected using the luminescence-based assay (Fig. 1*B*). Additionally, although it has been difficult to detect weak cellular responses to l-Asp using the fluorescence-based assay (17), the responses to l-Asp were successfully detected using the luminescence-based assay (Fig. 1*B*). Thus, we chose to use the luminescence-based assay for the following investigations.

To examine the response profiles to l-amino acids, HEK293T cells expressing the T1R1/T1R3 receptor were stimulated with 50 mm concentrations of each amino acid, and the luminescence intensities following the receptor activations were compared among 17 types of amino acids. A concentration of 50 mm was the upper limit for the l-Glu concentration in our luminescence assay due to its high osmotic pressure. In sensory tests of the 20 proteinogenic amino acids, humans perceived neutralized salts of l-Glu and l-Asp as having an umami taste (26). hT1R1/hT1R3 exhibited the highest response intensities to l-Glu and l-Asp of the 17 evaluated amino acids (Fig. 1*C*). hT1R1/hT1R3 also demonstrated slight but significant responses to l-Ala, l-Ser, l-Gln, l-Asn, l-Arg, and l-His (Fig. 1*C*). Of these amino acids, l-Ala, l-Ser, l-Gln, and l-Asn elicit a weak umami taste at high concentrations in human sensory tests (27). Because cellular responses for hT1R1/hT1R3 were subject to interference from the osmotic pressure of the sample solutions, the l-Arg and l-His responses for hT1R1/hT1R3 may result from the high osmotic pressure of the respective sample solutions (see “Experimental Procedures”). In contrast, the response intensities of mT1R1/mT1R3 to several types of l-amino acids were much higher than those to acidic amino acids, which is consistent with a previous report (Fig. 1*C*) (3). These results indicate that the sequence differences in these receptors influences the differences in ligand specificity between human and mouse T1R1/T1R3.

To determine which subunit is most important in defining the ligand specificity, the response patterns of mixed pairs of human and mouse T1Rs (*i.e.* hT1R1/mT1R3 or mT1R1/hT1R3) were examined. We selected four amino acids as representative ligands for human or mouse T1R1/T1R3: l-Glu and l-Asp for hT1R1/hT1R3 and l-Ala and l-Ser for mT1R1/mT1R3 (Fig. 1*C*). hT1R1/mT1R3 exhibited higher response intensities to acidic amino acids than to l-Ala and l-Ser (Fig. 1*D*), as was the case for hT1R1/hT1R3, which suggests that the T1R1 subunit is responsible for amino acid recognition. In contrast, mT1R1/hT1R3 did not respond to any amino acid tested (Fig. 1*D*). It has been reported that cells expressing mT1R2/hT1R3 also failed to respond to all evaluated sweeteners (19, 21).

To determine which domain of T1R1 is most critical for amino acid recognition, we investigated the response patterns of human and mouse chimeric T1R1 receptors by exchanging their VFTDs, which should contain the l-Glu binding site (16). hVFTD-mCRD-mTMD T1R1/mT1R3 exhibited higher response intensities to acidic amino acids than those to l-Ala and l-Ser, as did hT1R1/mT1R3 (Fig. 1*E*). In contrast, mVFTD-hCRD-hTMD T1R1/mT1R3 demonstrated higher response intensities to l-Ala and l-Ser than those to acidic amino acids (Fig. 1*F*). Additionally, mVFTD-hCRD-hTMD T1R1/mT1R3 exhibited higher response intensities to most of the other amino acids than to acidic amino acids, as did mT1R1/mT1R3 (Fig. 1*F*). These results suggest that the VFTD of T1R1 is critical for both human-type and mouse-type amino acid recognition.

**FIGURE 2.**
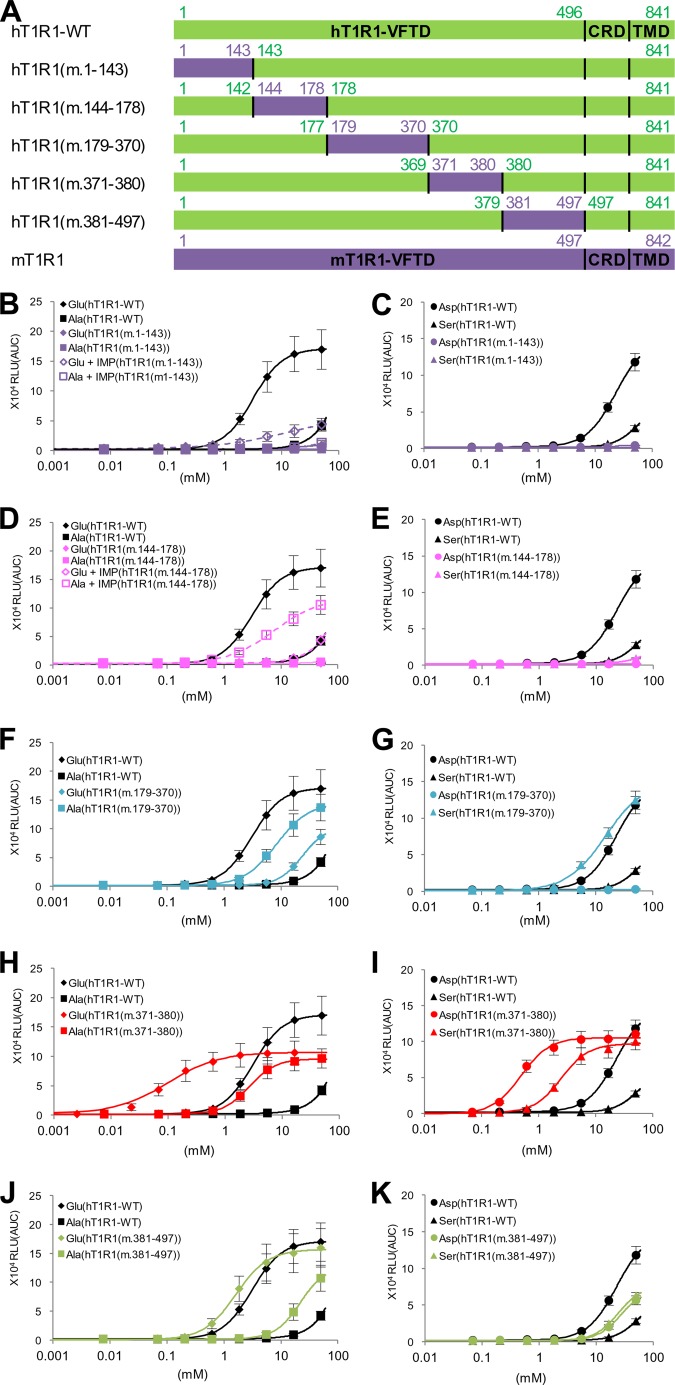
**The ligand specificity of human-to-mouse chimeric T1R1.** *A*, schematic diagrams of human-to-mouse chimeric T1R1 receptors. Regions from hT1R1 are shown in *green*, and regions from mT1R1 are shown in *purple*. hT1R1(m.1–143) comprised residues 1–143 from mT1R1 with the remaining sequence of hT1R1. hT1R1(m.144–178) comprised residues 1–142 from hT1R1, residues 144–178 from mT1R1, and residues 178–841 from hT1R1. The other chimeras follow an identical naming scheme. The alignments of the amino acid sequences are shown in Fig. 6*B. B–K*, critical regions in the VFTD of T1R1. Each human-to-mouse chimeric T1R1 was coexpressed with both mT1R3 and rG15i2, and the dose-response curves to l-Glu, l-Ala (*B*, *D*, *F*, *H*, and J), l-Asp, and l-Ser (*C*, *E*, *G*, *I*, and *K*) were examined in the absence (*solid lines with filled symbols*) or presence (*dashed lines with open symbols*) of 1 mm IMP. The values represent the mean ± S.E. of the RLU (AUC) of 5–6 recorded wells.

**FIGURE 3.**
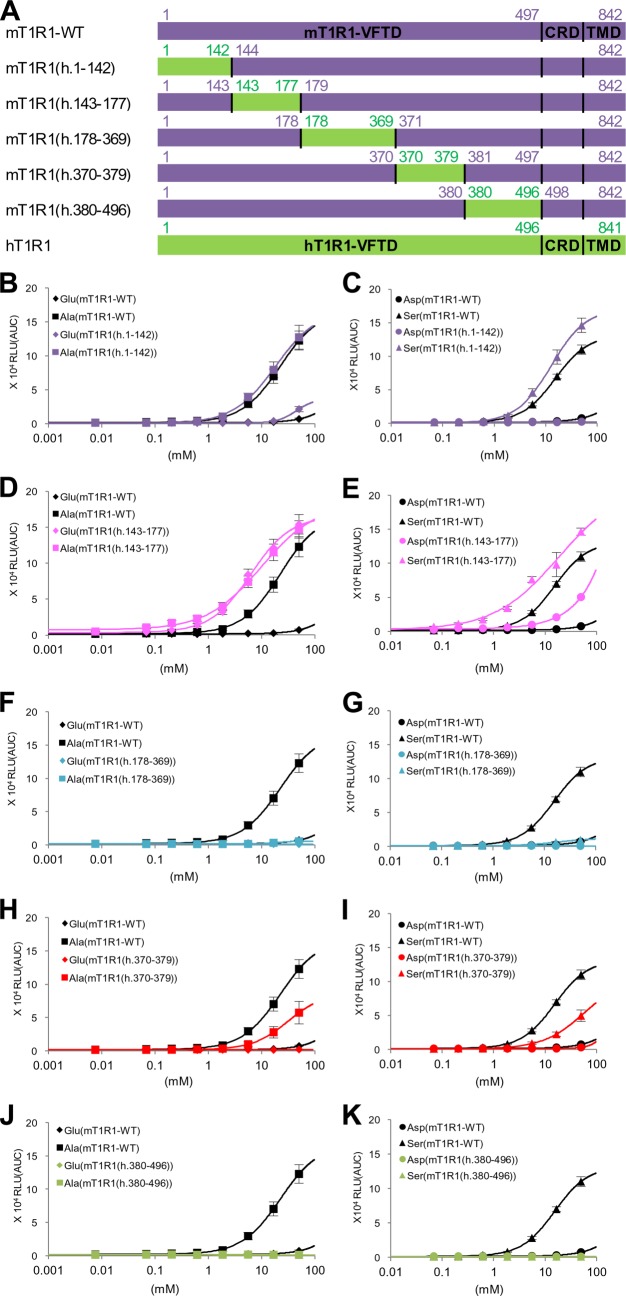
**The ligand specificity of mouse-to-human chimeric T1R1.** *A*, schematic diagrams of mouse-to-human chimeric T1R1 receptors. Regions from hT1R1 are shown in *green*, and regions from mT1R1 are shown in *purple*. The alignments of the amino acid sequences are shown in Fig. 6*B. B–K*, critical regions in the VFTD of T1R1. Each mouse-to-human chimeric T1R1 was coexpressed with both mT1R3 and rG15i2, and the dose-response curves to l-Glu, l-Ala (*B*, *D*, *F*, *H*, and *J*), l-Asp, and l-Ser (*C*, *E*, *G*, *I*, and *K*) were examined. The values represent the mean ± S.E. of the RLU (AUC) of six recorded wells.

To identify the region that is crucial for amino acid recognition more precisely, we constructed five human-to-mouse chimeric receptors encompassing the entire region of the VFTD of T1R1 (Fig. 2*A*). Dose-response curves for the representative four l-amino acids were examined, and the regions that affected the activity (potency and/or efficacy) of acidic amino acids were selected as the regions that are crucial for human-type responses, and the regions that affected the l-Ala and l-Ser activities were chosen as the regions that were important for the mouse-type responses. Of the five evaluated chimeric mutants, hT1R1(m.1–143) and hT1R1(m.144–178) demonstrated either slight or no responses to the four amino acids even at the highest evaluated concentration (50 mm) (Fig. 2, *B–E*). To evaluate the ligand specificity of these two receptors, we examined their responses to l-Glu and l-Ala in the presence of IMP because it has been reported that the responses of T1R1/T1R3 to various l-amino acids are dramatically enhanced by the addition of IMP (3, 16). In the presence of IMP, both l-Glu and l-Ala responses for hT1R1(m.144–178) were potentiated, but the l-Glu activity was lower than the l-Ala activity (Fig. 2*D*). These results suggest that hT1R1(m.144–178) retained its receptor function, but the introduction of mouse-type mutations in this region severely reduced the activity of acidic amino acids. Among the reverse set of chimeric receptors (Fig. 3), mT1R1(h.143–177) exhibited remarkable increases in the activity of acidic amino acids, and l-Glu activity was as high as the l-Ala activity (Fig. 3, *D* and *E*). These results suggested that the crucial residues for acidic amino acid recognition lie within residues 143–177 in hT1R1. In contrast, the l-Glu activity for hT1R1(m.1–143) was higher than the l-Ala activity in the presence of IMP, as was observed for hT1R1-WT (Fig. 2*B*). The response intensities to 50 mm l-Glu for mT1R1(h.1–142) were higher than those for mT1R1-WT (Fig. 3*B*), but the l-Asp responses for this receptor were lower than those for mT1R1-WT at several evaluated concentrations (Fig. 3*C*). The substitution of residues 1–142 in hT1R1 to the corresponding mouse residues (residues 1–143) may have affected the functional expression of the receptors (*e.g.* the maintenance of the overall conformation of T1R1/T1R3 or the cell-surface targeting of the receptors) rather than the specific recognition of acidic amino acids. hT1R1(m.179–370) exhibited decreases in the activity of acidic amino acids, whereas it exhibited increases in the l-Ala and l-Ser activity (Fig. 2, *F* and *G*). hT1R1(m.381–497) exhibited decreases in the l-Asp activity, whereas it exhibited increases in the l-Ala and l-Ser activity (Fig. 2, *J* and *K*). These results indicate that the regions corresponding to residues 179–370 and 381–497 in mT1R1 (residues 178–369 and 380–496 in hT1R1) include important residues for both human-type and mouse-type responses. The reverse chimeras for these two regions, *i.e.* mT1R1(h.178–369) and mT1R1(h.380–496), demonstrated either slight or no responses to the four evaluated l-amino acids (Fig. 3, *F*, *G*, *J*, and *K*). Because IMP barely potentiated the responses of these receptors to l-Glu and l-Ala (data not shown), the introduction of human-type mutations in these regions (residues 179–370 and 381–497 in mT1R1) may have affected the functional expression of mT1R1/mT1R3. The potency of l-Ala and l-Ser for hT1R1(m.371–380) was higher than that for hT1R1-WT (Fig. 2, *H* and *I*), indicating that the residues 371–380 in mT1R1 include those that are important for mouse-type responses. hT1R1(m.371–380) exhibited remarkable increases in the potency of not only the ligands of mT1R1/mT1R3 (l-Ala and l-Ser) but also acidic amino acids (the EC_50_ for l-Glu was 0.11 ± 0.01 mm for hT1R1(m.371–380) and 3.13 ± 0.19 mm for hT1R1-WT) (Fig. 2, *H* and *I*). Conversely, the reverse chimera mT1R1(h.370–379) demonstrated decreases in the activity of all four evaluated l-amino acids (Fig. 3, *H* and *I*).

We thus selected residues within the 143–177, 178–369, and 380–496 regions of hT1R1 to screen for residues that may be critical for acidic amino acid recognition, whereas residues within the 179–370, 371–380, and 381–497 regions of mT1R1 were selected to screen for residues that may be critical for the mouse-type broad response.

##### Critical Residues for Acidic Amino Acid Recognition

**FIGURE 4.**
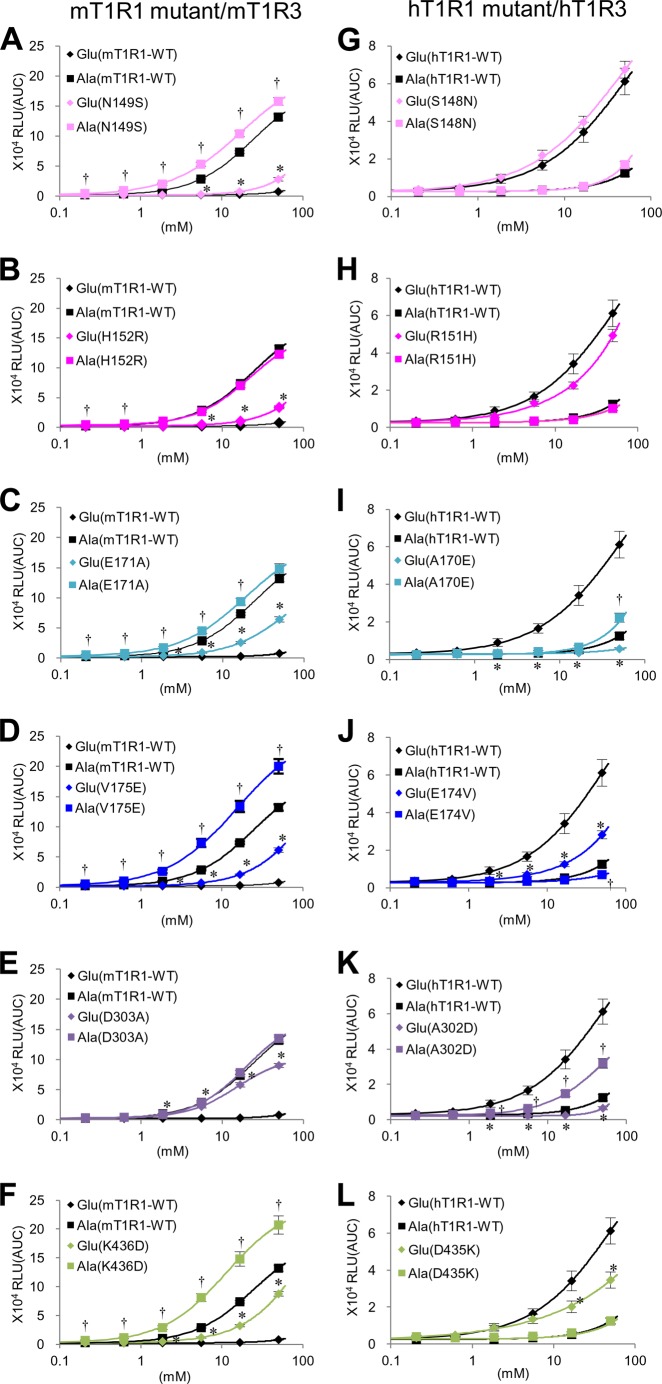
**Residues that are critical for acidic amino acid recognition.** *A–F*, point mutants in the VFTD of mT1R1 demonstrated increased responses to l-Glu. Each WT and mutant mT1R1 was transfected into HEK293T cells together with mT1R3 and rG15i2, and the dose-response curves to l-Glu and l-Ala were determined using a luminescence-based assay. *G–L*, dose-response curves of the hT1R1-mutants and WT to l-Glu and l-Ala. Each WT and mutant hT1R1 was transfected into HEK293T cells together with hT1R3 and rG15i2. The values represent the mean ± S.E. of the RLU (AUC) of 6–7 recorded wells. Significant differences between WT (*A–F*, mT1R1-WT; *G–L*, hT1R1-WT) and mutant receptors were analyzed using Student's *t* test (*, *p* < 0.05 for l-Glu responses; †, *p* < 0.05 for l-Ala responses).

To identify the residues that are critical for human-type acidic amino acid recognition, additional mouse-to-human T1R1 chimeras and T1R1 point mutants (see “Experimental Procedures”) were constructed involving residues 143–369 and 380–496 in hT1R1 because these regions were shown to contain the residues that are critical for human-type responses in the previous studies (Figs. 2 and 3). These receptors were examined for their response patterns to the four representative l-amino acids, and we identified the set of residues that influenced the response to acidic amino acids. Among the point mutants in these regions, the following six mutants that exhibited increases in the l-Glu responses were identified: mT1R1-N149S, -H152R, -E171A, -V175E, -D303A, and -K436D (Fig. 4, *A–F*). These six mutants demonstrated higher responses to l-Glu than those for mT1R1-WT at several evaluated concentrations (Fig. 4, *A–F*). These results suggest that Ser-148, Arg-151, Ala-170, Glu-174, Ala-302, and Asp-435 in hT1R1 contributed to the changes in the acidic amino acid responses for the human-mouse chimeric receptors in the previous studies (Figs. 2 and 3). To determine whether any of these six residues played an essential role in hT1R1/hT1R3 function, we generated hT1R1 point mutants of these residues (Fig. 4, *G–L*). Of the six mutant receptors, hT1R1-A170E and -A302D did not exhibit detectable responses to l-Glu up to the highest evaluated concentration (50 mm) (Fig. 4, *I* and *K*). Conversely, these mutations did not result in decreases in the l-Ala responses in comparison to hT1R1-WT (Fig. 4, *I* and *K*), which indicates that the mutations A170E and A302D selectively reduced the l-Glu activities. hT1R1-E174V and -D435K exhibited lower responses to l-Glu than those for hT1R1-WT at several evaluated concentrations (Fig. 4, *J* and *L*). Because the mutation D435K in hT1R1 did not affect the l-Ala responses, its functional expression was confirmed (Fig. 4*L*). Conversely, the E174V mutation resulted in decreases in both the l-Glu and the l-Ala responses (Fig. 4*J*). The E174V mutation should affect the general amino acid recognition (or the functional expression of receptors) rather than the specific acidic amino acid responses. hT1R1-S148N and -R151H exhibited little direct effect on the l-Glu and l-Ala activity (Fig. 4, *G* and *H*). These results suggest that of the six residues, Ala-170 and Ala-302 in hT1R1 (Glu-171 and Asp-303 in mT1R1) are the most critical residues for acidic amino acid recognition.

##### Key Residues for the Broad l-Amino Acid Response

**FIGURE 5.**
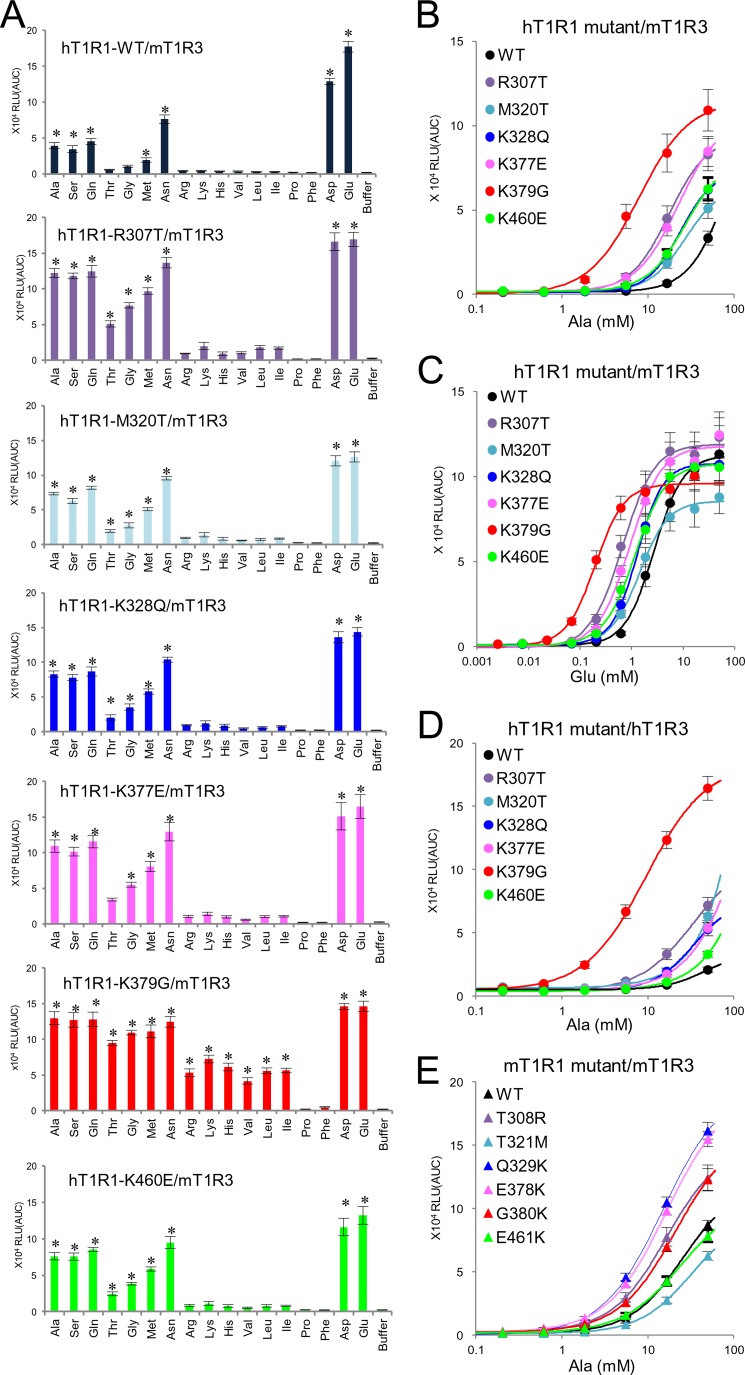
**Residues that are critical for the broadly tuned response to l-amino acids.** *A*, response profiles to 17 amino acids for hT1R1 mutants, which exhibited increased activity to various amino acids. Each WT and hT1R1-mutant was transfected into HEK293T cells together with mT1R3 and rG15i2, and 50 mm concentrations of each amino acid were applied. The values represent the mean ± S.E. of the RLU (AUC) of 5–6 recorded wells. Significant differences from the responses to buffer were analyzed using one-way ANOVA followed by Dunnett's test (*p < 0.05). *B–D*, each WT and mutant hT1R1 was transfected into HEK293T cells together with mT1R3 (*B* and *C*) or hT1R3 (*D*), and the dose-response curves to l-Ala (*B* and *D*) and l-Glu (*C*) were examined. *E*, the dose-response curves of mT1R1 mutants to l-Ala. Each WT and mutant mT1R1 was transfected into HEK293T cells together with mT1R3 and rG15i2. The values represent the mean ± S.E. of the RLU (AUC) of 5–8 recorded wells.

To better identify the residues responsible for mouse-type responses, we constructed additional human-to-mouse chimeric receptors of T1R1 and point mutants within residues 179–497 in mT1R1 (see “Experimental Procedures”) because this region should contain the residues that are critical for l-Ala and l-Ser recognition (Figs. 2 and 3). These receptors were coexpressed with mT1R3, and their response patterns to the four representative l-amino acids were compared with the response pattern of hT1R1-WT. Six single point mutations in hT1R1 that enhanced the l-Ala and l-Ser responses were identified: R307T, M320T, K328Q, K377E, K379G, and K460E (Fig. 5, *A* and *B*). To confirm whether these residues were related to the broadly tuned response of mT1R1/mT1R3, the response patterns of these hT1R1 mutants to 17 amino acids were examined. These six hT1R1 mutants exhibited detectable responses to various amino acids, some of which were not detected for hT1R1/mT1R3 (Fig. 5*A*). These results suggest that these six residues are responsible for the mouse-type, broadly tuned response. Notably, although mT1R1/mT1R3 only weakly recognizes acidic amino acids, increases in the potency of l-Glu were found for all six hT1R1 mutants in comparison to hT1R1-WT (Fig. 5*C* and Table 1), as was observed for the chimeric receptor hT1R1(m.371–380) (Fig. 2*H*). Even when coexpressed with hT1R3, all of the hT1R1 mutants exhibited increased responses to l-Ala at several evaluated concentrations (Fig. 5*D*). Of the six mutations in hT1R1, the K379G mutation resulted in the greatest increases in the activity of most of the evaluated 17 amino acids (Fig. 5, *A–D*). However, among the reverse mutants, only mT1R1-T321M exhibited lower responses to l-Ala in comparison to mT1R1-WT at several evaluated concentrations, and mT1R1-G380K did not exhibit reduced activity in response to l-Ala (Fig. 5*E*). These results suggest that the mouse-type responses should be retained with the cooperation of multiple residues, including these six residues.

**TABLE 1:** **The effect of mouse-type mutations on l-Glu responses** The EC_50_ and *E*_max_ values for the l-Glu-induced response in HEK293T cells transiently transfected with the hT1R1-WT or mutant receptors together with mT1R3 and rG15i2 are shown. The results are presented as the mean ± S.E. of 5–7 dose-response measurements. Significant differences between WT and mutant receptors using one-way ANOVA followed by Dunnett's test are indicate by the footnotes.

hT1R1	Glu	
	EC_50_	*E*_max_
	*mm*	× *10^4^ RLU (AUC)*
WT	2.7 ± 0.4	11.2 ± 1.6
R307T	0.7 ± 0.1*^a^*	12.0 ± 1.3
M320T	1.4 ± 0.1*^b^*	8.6 ± 1.0
K328Q	1.3 ± 0.1*^b^*	10.8 ± 1.0
K377E	0.9 ± 0.03*^b^*	11.9 ± 1.2
K379G	0.2 ± 0.01*^a^*	9.6 ± 1.0
K460E	1.2 ± 0.1*^b^*	10.7 ± 0.7

*^a^ p* < 0.001.

*^b^ p* < 0.01.

##### Two Distinct Determinants of the Amino Acid Recognition of T1R1

**FIGURE 6.**
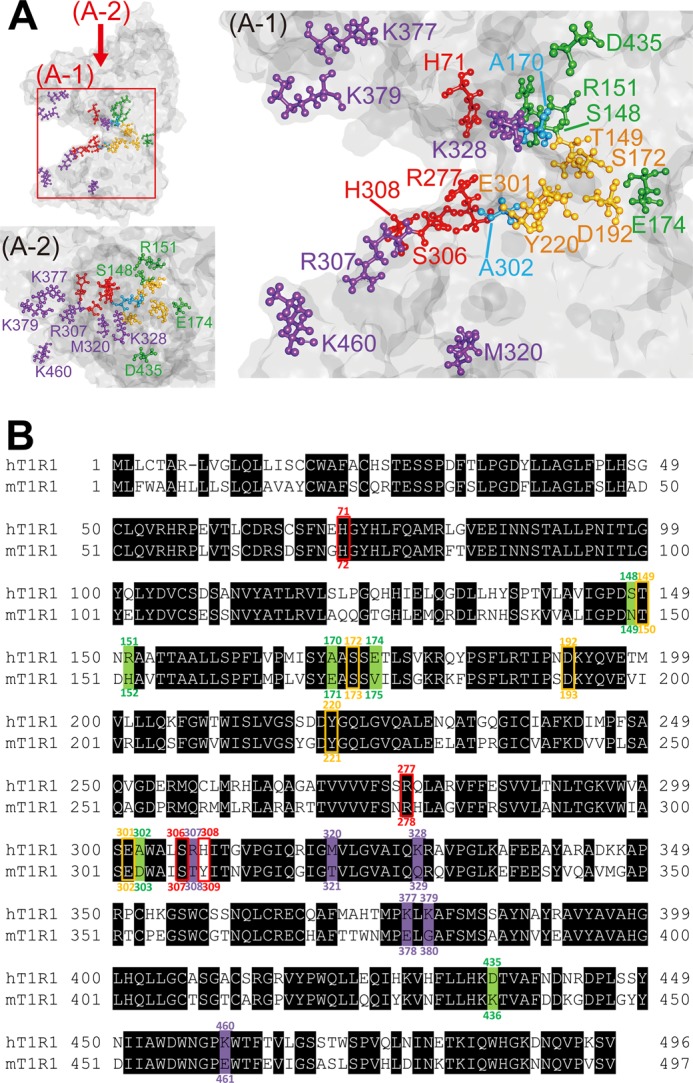
**Two distinct determinants of amino acid selectivity in T1R1.** *A*, mapping of the critical residues on a molecular model of the VFTD of hT1R1. The models are oriented in the horizontal (*A-1*) and vertical (*A-2*) directions. The hinge region is to the *right*, and the opening is to the *left*. The six residues that are critical for acidic amino acid recognition that were identified in this study are colored *green* (Ser-148, Arg-151, Glu-174, and Asp-435) and *cyan* (Ala-170 and Ala-302). The six residues that are critical for the broadly tuned responses to l-amino acids (Arg-307, Met-320, Lys-328, Lys-377, Lys-379, and Lys-460) are colored *purple*. The residues that were reported to be critical for l-Glu binding based on a previous study (16) (Thr-149, Ser-172, Asp-192, Tyr-220, and Glu-301) are colored *yellow*, and those that were reported to be critical for IMP binding (His-71, Arg-277, Ser-306, and His-308) are colored *red* (16). *B*, sequence alignment of the VFTD of human and mouse T1R1. The six residues that are critical for acidic amino acid recognition that were identified in this study are colored *green*. The six residues that are critical for the broadly tuned responses to l-amino acids are colored *purple*. The residues that were reported to be critical for l-Glu binding based on a previous study (16) are colored *yellow*, and the residues that were reported to be critical for IMP binding (16) are colored *red*.

We mapped each of the six key residues for the human-type and mouse-type responses onto a molecular model of the VFTD of hT1R1, which was built using the open-form structures of mGluR1 (Fig. 6*A*) (13). Of the six key residues that are involved in acidic amino acid recognition, five residues, *i.e.* Ser-148, Arg-151, Ala-170, Glu-174, and Ala-302, are positioned in the hinge region near the residues that were identified as critical for l-Glu binding in a previous site-directed mutagenesis study (16). In particular, the two most critical residues, Ala-170 and Ala-302 (Fig. 4), are paired at the edges of the upper and lower lobes of the l-Glu binding site, respectively. The remaining residue, Asp-435, is located outside of the hinge region (Fig. 6*A-2*).

**FIGURE 7.**
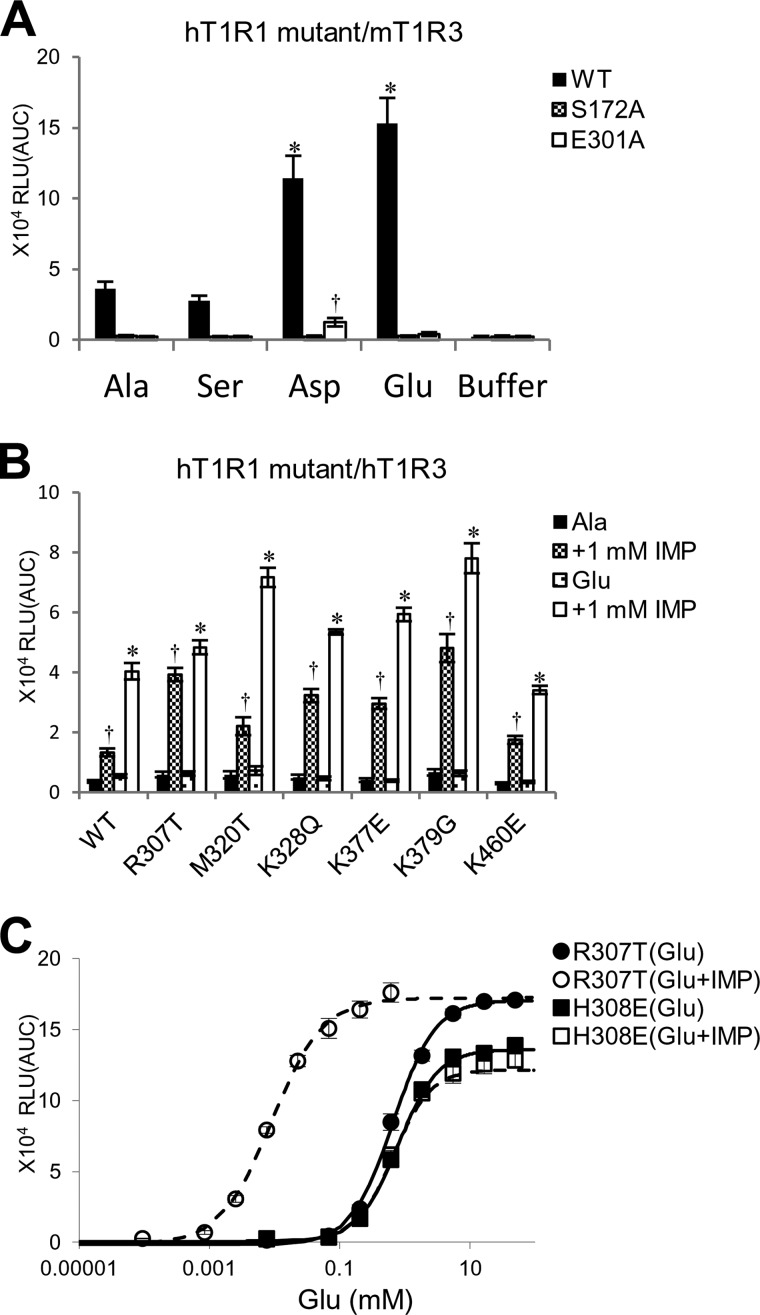
*A*, hT1R1 mutants in the l-Glu binding site exhibited weaker responses to l-Ala, l-Ser, and acidic amino acids. HEK293T cells were transfected with each hT1R1 mutant and WT together with mT1R3 and rG15i2 and stimulated with 50 mm concentrations of each amino acid. The values represent the mean ± S.E. of the RLU (AUC) of four recorded wells. Significant differences from the response to buffer were analyzed using one-way ANOVA followed by Dunnett's test (*, *p* < 0.05 for hT1R1-WT; †, *p* < 0.05 for hT1R1-S172A). *B* and *C*, hT1R1 mutants in the residues that are critical for the broadly tuned response retained the enhanced activity in response to IMP. *B*, the IMP-enhanced activities of hT1R1 mutants that gained the ability to induce broadly tuned responses. Cells expressing each of the hT1R1 mutants together with hT1R3 and rG15i2 were stimulated with l-Ala or l-Glu in the absence or presence of 1 mm IMP. The l-Glu concentrations used in the assay were 1 mm (WT), 0.1 mm (R307T), 0.2 mm (M320T, K328Q, K377E, and K460E), and 0.01 mm (K379G) mm, whereas the Ala concentrations used were 15 mm (WT), 5 mm (Arg-307), 10 mm (M320T, K328Q, K377E, and K460E), and 1 mm (K379G) mm. Significant differences between the amino acid responses with and without IMP were analyzed using Student's *t* test (*, *p* < 0.05 for l-Glu; †, *p* < 0.05 for l-Ala). *C*, IMP activities of hT1R1 mutants of either the IMP-binding site (H308E) or the residue that is critical for the broadly tuned response (R307T). The activity of hT1R1-R307T was enhanced by IMP (the EC_50_ values were 0.7 ± 0.1 mm for l-Glu and 0.008 ± 0.002 mm for l-Glu + IMP), whereas the activity of H308E was not enhanced by IMP (the EC_50_ values are 0.7 ± 0.1 mm for l-Glu and 0.8 ± 0.1 mm for l-Glu + IMP). Each of the hT1R1 mutants was coexpressed with both mT1R3 and rG15i2, and the dose-response curves to l-Glu were evaluated in the presence and absence of 1 mm IMP. The values represent the mean ± S.E. of the RLU (AUC) of 5–7 recorded wells.

In contrast, all of the residues that are critical for the broadly tuned response lie in regions that are distinct from the orthosteric binding site (Fig. 6*A*). Of the six key residues, four (Arg-307, Lys-377, Lys-379, and Lys-460) are positioned on the outer side of the predicted IMP binding region (16) near the opening of the VFTD. Another residue, Met-320, is located at the internal side of the lower lobe, and the final residue, Lys-328, is located outside of the hinge region. To clarify whether l-amino acids other than l-Glu bind at the reported l-Glu binding site or at the newly identified non-orthosteric site, we examined the response patterns to the four representative l-amino acids of two hT1R1-mutants, hT1R1-S172A and -E301A. These two mutants contained mutations at residues that are reportedly critical for l-Glu binding (Fig. 6), and these mutations reduced the activity in response to l-Glu (16). Consequently, neither hT1R1-S172A nor -E301A demonstrated detectable responses to l-Ala or l-Ser (Fig. 7*A*), suggesting that l-amino acids other than l-Glu also bind at the l-Glu binding site in the hinge region. Collectively with the results that the introduction of mouse-type mutations in the six key residues for mouse-type responses conferred higher activities of mT1R1/mT1R3 ligands and acidic amino acids for the hT1R1 receptor (Fig. 5*C* and Table 1), the residues that were critical for broadly tuned responses should be related to the modulation of receptor activity rather than to the binding of l-amino acids.

Because some residues that were critical for broadly tuned responses were located near the reported IMP-binding site (Fig. 6*A*), we examined whether the key residues for the broadly tuned responses modulated the receptor activity in an identical manner as the enhancement via IMP. We compared the effect of IMP application on the l-amino acid responses among the six hT1R1 mutants in the newly identified non-orthosteric sites and a hT1R1 mutant in the IMP-binding site (hT1R1-H308E). hT1R1-H308E was introduced as a reverse charge mutation in the IMP-binding site, which partly mimicked the stable closed conformation that is induced upon IMP binding (16). All six mutants that exhibited high receptor activity in this study retained the synergistic effect between l-amino acids and IMP (Fig. 7, *B* and *C*, *R307T*). Conversely, the receptor activity of hT1R1-H308E was no longer enhanced upon the addition of IMP, as reported in a previous study (Fig. 7*C*) (16). These results suggest that the residues that are critical for broadly tuned responses modulate the receptor activity in a manner that is distinct from that of IMP.

Based on our site-directed mutagenesis analysis and molecular modeling results, we hypothesized that the ligand specificity of T1R1 is determined by a combination of two distinct factors. 1) the amino acid selectivity, which is characterized by the residues at the orthosteric binding site, and 2) the receptor activity, which is modulated by residues at the non-orthosteric site.

**FIGURE 8.**
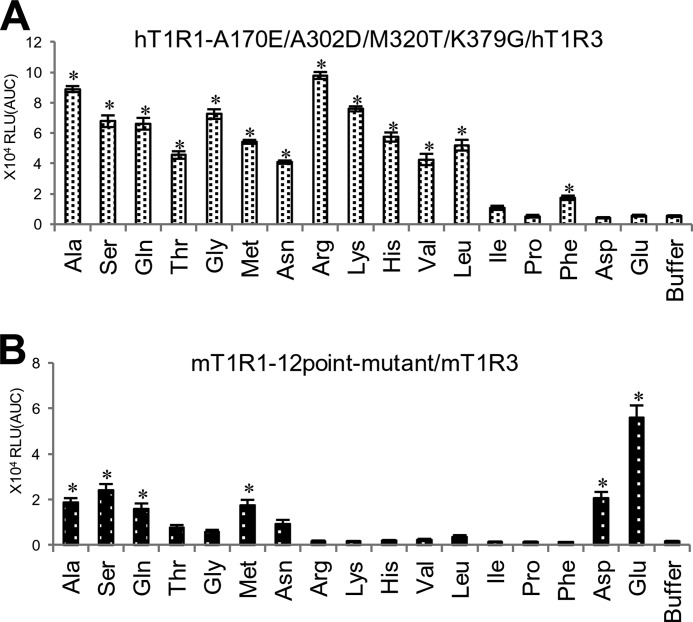
**The response profiles of the multiple point mutants to 17 amino acids.** The multiple point mutants included mutations in residues that are critical for both acidic amino acid recognition and the broadly tuned response. HEK293T cells were transfected with hT1R1-A170E/A302D/M320T/K379G/hT1R3 (*A*) or mT1R1-N149S/H152R/E171A/V175E/D303A/T308R/T321M/Q329K/E378K/G380K/K436D/E461K/mT1R3 (*B*) together with rG15i2 and then stimulated with 50 mm concentrations of each amino acid. The values represent the mean ± S.E. of the RLU (AUC) of six recorded wells. Significant differences from the response to buffer were analyzed using one-way ANOVA followed by Dunnett's test (*, *p* < 0.05).

To confirm this hypothesis, we constructed multiple point mutants for both determinants in hT1R1 and mT1R1 and evaluated whether the ligand specificities of these receptors were similar to those of the T1R1s of other species. First, we generated a quadruple mutant hT1R1 in which each of the residues that is critical for acidic amino acid recognition (Ala-170 and Ala-302 in hT1R1; Fig. 4) and broadly tuned responses (Met-320 and Lys-379 in hT1R1; Fig. 5) was mutated to the corresponding mouse residues. The mutant hT1R1-A170E/A302D/M320T/K379G exhibited higher response intensities to various amino acids than to acidic amino acids, as was observed for mT1R1-WT when the response intensities to 50 mm concentrations of each amino acid were compared among 17 types of amino acids (Fig. 8*A*). In contrast, the 12-point mutant mT1R1, in which all 12 key residues were mutated to the corresponding human residues, exhibited the highest response intensity to l-Glu among 17 evaluated amino acids, as was observed for hT1R1-WT (Fig. 8*B*). These results confirm the validity of our hypothesis that a combination of these two factors determines the ligand specificity of T1R1/T1R3.

##### Characteristics of Nonhuman Primate T1R1

Sensitivity to l-Glu varies among primate species (28). Expecting that altering key determinant residues of T1R1 would modify their sensitivity to l-amino acids, we compared the deduced coding sequences of T1R1 from three nonhuman primate species and examined the response patterns of the encoded receptors. We selected two species of Old World monkeys (the rhesus macaque, *Macaca mulatta*, and the hamadryas baboon, *Papio hamadryas*) and one species of New World monkeys (the Bolivian squirrel monkey, *Saimiri boliviensis*).

**FIGURE 9.**
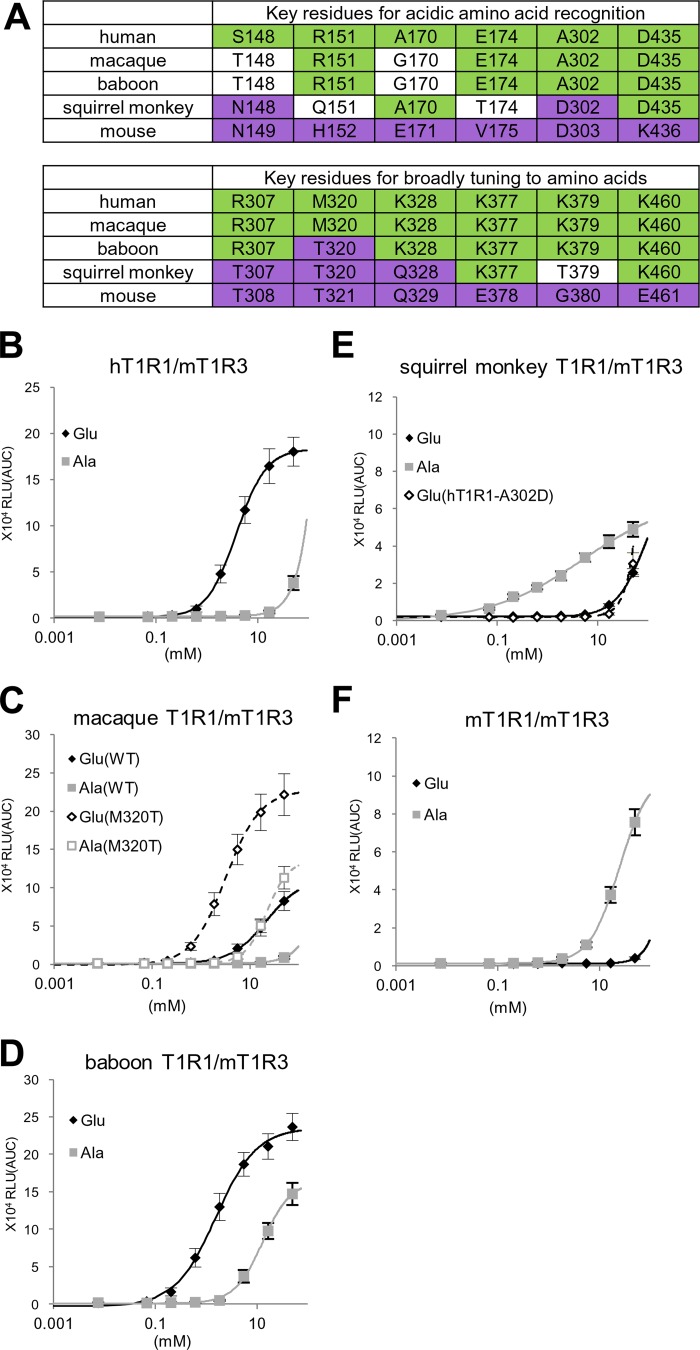
**Twelve critical residues and the amino acid responses of T1R1 in nonhuman primates.** *A*, the 12 residues that are critical for acidic amino acid recognition and the broadly tuned response of human and mouse T1R1 and the equivalent residues of T1R1 in nonhuman primates. Residues that are conserved with hT1R1 are colored *green*, and residues that are conserved with mT1R1 are colored *purple. B–F*, the dose-response curves of human (*B*), macaque (*C*), baboon (*D*), squirrel monkey (*E*), and mouse (*F*) T1R1/mT1R3 to l-Glu and l-Ala. The results obtained for mutants (macaque T1R1-M320T and hT1R1-A302D) are also shown in *C* and *E*, respectively (*dashed lines with open symbols*). The values represent the mean ± S.E. of the RLU (AUC) of five recorded wells. The EC_50_ values of l-Glu are 3.3 ± 0.4 mm for macaque T1R1-M320T and 1.8 ± 0.4 mm for baboon T1R1.

When coexpressed with mT1R3, the macaque T1R1, in which all 12 critical residues are identical (10 residues) or similar (2 residues) to those in hT1R1 (Fig. 9*A*), exhibited higher l-Glu activity than l-Ala activity, as with hT1R1 (Fig. 9, *B* and *C*). Baboon T1R1, in which all key residues except for Thr-320 were identical to those in macaque T1R1 (Fig. 9*A*), also exhibited higher l-Glu activity than l-Ala activity (Fig. 9*D*). Moreover, baboon T1R1 exhibited higher l-Glu and l-Ala activities than those for macaque T1R1 (Fig. 9, *C* and *D*). The introduction of the mutation M320T into macaque T1R1 resulted in increases in both l-Glu and l-Ala activities (Fig. 9*C*), demonstrating that Thr-320 contributes to increased receptor activity in baboon T1R1.

In contrast, in the squirrel monkey T1R1, 5 of the 12 key residues are identical to those in mT1R1 (Fig. 9*A*). Notably, one of the residues that is critical for acidic amino acid recognition (Asp-302) is identical to that in mT1R1. Additionally, of the six key residues that are important for the broadly tuned response, three are conserved (Thr-307, Thr-320, and Gln-328), whereas Thr-379 is similar to the corresponding residue in mT1R1 (Gly-380). As expected from the amino acid sequence, the l-Glu activity for the squirrel monkey T1R1 was as low as that for the hT1R1-A302D mutant receptor (Fig. 9*E*). Conversely, the squirrel monkey T1R1 demonstrated the greatest l-Ala potency among the four primate T1R1 receptors (Fig. 9, *B–E*). These results reinforce the importance of the 12 residues that were identified in this study.

## DISCUSSION

In this study we investigated the molecular mechanism underlying species-dependent differences in the ligand specificity of T1R1/T1R3. Site-directed mutagenesis analysis and molecular modeling indicated that the human-type acidic amino acid recognition and mouse-type broadly tuned response are controlled by separate sets of amino acids in the VFTD of T1R1 (Fig. 6).

### 

#### 

##### The Orthosteric Site Is Responsible for Human-type Acidic Amino Acid Recognition

The l-Glu binding site of hT1R1 has been previously proposed to lie within the hinge region of the VFTD based on site-directed mutagenesis analysis and molecular models that are based on the structures of mGluRs (15, 16, 29). However, the crucial determinants of the l-Glu-specific response in human receptors have not yet been examined. In this study, six residues that contributed to the acidic amino acid responses have been identified: Ser-148, Arg-151, Ala-170, Glu-174, Ala-302, and Asp-435 in hT1R1 (Fig. 4). Of the six residues, five (Ser-148, Arg-151, Ala-170, Glu-174, and Ala-302) are located in the hinge region (Fig. 6*A*). Additionally, Ser-148, Arg-151, Ala-170, and Ala-302 reportedly correspond to the l-Glu binding site in mGluR1 (15, 30), indicating that acidic amino acid recognition is primarily attributable to the properties of the orthosteric ligand binding site. One marked difference between hT1R1 and mT1R1 lies in the electrostatic potential profile of this site. Of the six key residues, four are negatively charged in either hT1R1 or mT1R1 (*i.e.* h/m; Ala-170/Glu-171, Glu-174/Val-175, Ala-302/Asp-303, and Asp-435/Lys-436) (Fig. 6*B*). Of the mT1R1 mutants in which one of these six residues was mutated, four involving negatively charged residues (*i.e.* mT1R1-E171A, -V175E, -D303A, and -K436D) exhibited greater effects than the other two mutants (*i.e.* mT1R1-N149S and -H152R) (Fig. 4). In particular, the two most critical residues in hT1R1, Ala-170 and Ala-302 (Glu-171 and Asp-303 in mT1R1), are located in pairs at the edges of the upper and lower lobes of the orthosteric binding site, respectively (Fig. 6*A*). Ser-186 in mGluR1, which corresponds to Ala-170 in hT1R1, is known to interact with the distal carboxylic acid moiety of l-Glu via a water molecule (13, 30). Additionally, Gly-319 in mGluR1, which corresponds to Ala-302 in hT1R1, is also positioned near the carboxylate side chain of l-Glu (30, 31). In mT1R1, mutating Ala to an acidic residue at this position is certainly expected to affect acidic amino acid binding due to the electrostatic repulsion between the negative charges of the carboxylic acid moieties.

##### Non-orthosteric Sites Responsible for Mouse-type Broadly Tuned Responses to l-Amino Acids

In contrast to the recognition mechanism for l-Glu binding, the recognition mechanism for other l-amino acids is unknown. We identified six residues that are responsible for the mouse-type broadly tuned response (Fig. 5). The molecular model indicated that all six residues lie in regions that are distinct from the orthosteric binding site (Fig. 6*A*). The T1R family possesses multiple ligand binding sites in addition to its orthosteric binding domain (32). Therefore, it is possible that these residues are related to a novel amino acid binding site. However, these residues modulated the activities of not only the ligands of mT1R1/mT1R3 but also acidic amino acids, which are assumed to bind at the orthosteric binding site (Fig. 5, *B–D*). Moreover, we examined whether l-Ala and l-Ser bind at the orthosteric binding site using two hT1R1 mutants, hT1R1-S172A and -E301A, in which residues that are reportedly critical for l-Glu binding in the hinge region were mutated to an Ala residue (Fig. 7*A*) (16). Although hT1R1-S172A and -E301A reportedly retained their responses to the ligand “S807,” which interacts with the TMD of T1R1 (16), neither receptor exhibited a response to l-Ala or l-Ser (Fig. 7*A*). These results suggest that various l-amino acids bind at the orthosteric binding site and that the key residues for broadly tuned responses are related to the modulation of receptor activity. The mT1R1/mT1R3 receptor should thus exhibit broadly tuned responses to various amino acids because of its high receptor activity. We propose at least two possible mechanisms through which the receptor activity could be modulated; 1) the key residues for broadly tuned responses regulate the potencies of orthosteric ligands (l-amino acids) by inducing a conformational change that affects the association and/or dissociation rate of ligands at the orthosteric site (affinity modulation), and/or 2) these residues affect the signaling capacity after the binding of the amino acid to the orthosteric binding site (efficacy modulation) (33). Zhang *et al.* (34) previously proposed the existence of “pincer residues” near the opening of the VFTD and suggested that such pincer residues could be involved in lobe-lobe or lobe-enhancer interactions to help stabilize the closed conformation of T1Rs. For example, the enhancement activity of IMP may be induced by its coordination of the positively charged pincer residues via its negatively charged phosphate group to stabilize the closed conformation of the VFTD of T1R1 (16). Therefore, the introduction of a reverse charge mutation in the IMP-binding site of hT1R1 (H308E) conferred the receptor with greater l-Glu potency and efficacy, in part by mimicking the enhancement mechanism of IMP (16). However, all mutants that exhibited high receptor activity in this study retained the synergistic effect between l-amino acids and IMP (Fig. 7, *B* and *C*, *R307T*), whereas the activity of hT1R1-H308E was no longer enhanced by the addition of IMP, as reported in a previous study (Fig. 7*C*) (16). These results suggest that the residues that are critical for broadly tuned responses modulate the receptor activity in a manner that is distinct from that of IMP. Using molecular modeling, Roura *et al.* (29) proposed that Arg-307 in hT1R1 was critical for amino acid recognition and suggested that the presence of Thr (which is a neutral polar residue) in rodent T1R1 at this position rather than Arg (which is a charged polar residue) allows a wider range of l-amino acids to enter and interact with the orthosteric ligand binding site. Note that all five key residues on the surface of the VFTD differ in charge between hT1R1 and mT1R1 (*i.e.* h/m; Arg-307/Thr-308, Lys-328/Gln-329, Lys-377/E378, Lys-379/Gly-380, and Lys-460/Glu-461) (Fig. 6). Although the mechanism through which these residues enhance the receptor activity remains unclear, the electrostatic properties of the non-orthosteric site likely play an important role in the ligand specificity of T1R1, as was observed for the orthosteric binding site. Conversely, the other key residue, 320, which is located at the internal side of the lower lobe, modulated the l-amino acid activities without altering the electrostatic properties (Figs. 5 and 9*C*). The receptor activity of T1R1/T1R3 should be determined by multiple residues that regulate the potency and/or efficacy of the orthosteric ligands through various mechanisms, including the modulation of the local or general conformation of receptors from the side of the non-orthosteric site.

##### Determinants of the Ligand Specificity of T1R1/T1R3

Multiple point mutants for both determinants experimentally validated our hypothesis that the ligand specificity of T1R1/T1R3 is determined by a combination of two distinct factors; 1) the amino acid selectivity, which is characterized by the residues at the orthosteric binding site, and 2) the receptor activity, which is modulated by the residues at the non-orthosteric sites (Fig. 8). Additionally, we confirmed that the hypothesis regarding the existence of two determinants is also applicable to the T1R1 receptors of three species of nonhuman primates (Fig. 9). Although we cannot ignore the possibility that an introduction of a mutation affects the expression levels of the receptors rather than the efficacy of the ligands, these results validated the interpretation of the results from chimeric receptors and point mutants. Because the receptor activity of hT1R1/mT1R3 was higher than that of hT1R1/hT1R3, we propose that the residues that are critical for the receptor activities should lie at various non-orthosteric sites, including T1R3. Mammalian T1R1/T1R3 should vary in ligand specificity due to changes in the properties of both the orthosteric and non-orthosteric sites of T1R1/T1R3.

Recent studies have revealed that T1Rs and their downstream molecules (*e.g.* Gαgust, PLCβ2, and TRPM5) are widely distributed in a variety of organs (35–37). Although the function of T1R1/T1R3 in non-taste tissues remains unclear, the difference in ligand specificity between species should also affect physiological events other than taste perception. When and why did the changes in the ligands of T1R1/T1R3 occur? In this study we have elucidated how human T1R1 is specific for acidic amino acids. The identification of the residues that are crucial for amino acid recognition should provide a clue to reveal the evolutionary and physiological importance of changes in the ligands of T1R1/T1R3.

## Footnotes

2 The abbreviations used are:

mGluR: metabotropic glutamate receptor
VFTD: Venus flytrap domain
CRD: cysteine-rich domain
TMD: seven-transmembrane domain
AUC: area under the curve
RLU: relative light unit
ANOVA: analysis of variance.
